# Acute Massive Gastric Dilatation and Gastric Perforation as a Result of Closed-Loop Obstruction of the Stomach

**DOI:** 10.7759/cureus.13365

**Published:** 2021-02-16

**Authors:** Oseen Shaikh, Suresh Chilaka, Nikhil Reddy, Chellappa Vijayakumar, Uday Kumbhar

**Affiliations:** 1 Surgery, Jawaharlal Institute of Postgraduate Medical Education and Research, Puducherry, IND

**Keywords:** acute massive gastric dilatation, perforation, feeding jejunostomy, closed loop obstruction

## Abstract

Acute massive gastric dilatation is a rare event that is usually underdiagnosed. It can occur due to multiple etiologies, including medical and surgical, or as a postoperative complication. Acute massive gastric dilatation can lead to life-threatening fatal complications, including perforation, bleeding, or shock. We report a rare case of acute massive gastric dilatation with perforation of the stomach due to closed-loop obstruction of the stomach, which occurred in a patient with cricopharyngeal carcinoma due to a kink at the feeding jejunostomy site. Early diagnosis and treatment are essential, as acute massive gastric dilatation with perforation carries high morbidity and mortality.

## Introduction

Acute massive gastric dilatation (AMGD) is a rare disorder, missed many times, and can be a fatal event. The disease’s exact etiology is not very much known, but multiple factors have been proposed that can lead to AMGD. It can lead to severe and catastrophic complications such as gastric perforation, hemorrhage, bleeding, electrolyte disturbances, and shock, all of which carry a high mortality. We report a rare case of AMGD with gastric perforation occurring due to closed-loop obstruction of the stomach due to a kink at the feeding jejunostomy (FJ) site. The condition was detected early and managed promptly.

## Case presentation

A 30-year-old male patient presented with dysphagia and weight loss for two months and had absolute dysphagia for the last two weeks. The patient was diagnosed to have cricopharyngeal carcinoma (left pyriform fossa). The biopsy was suggestive of well-differentiated squamous cell carcinoma. Nasogastric tube (NGT) insertion was tried under fluoroscopy and endoscopic guidance but failed due to extensive growth. Hence, the patient underwent FJ.

**Figure 1:**
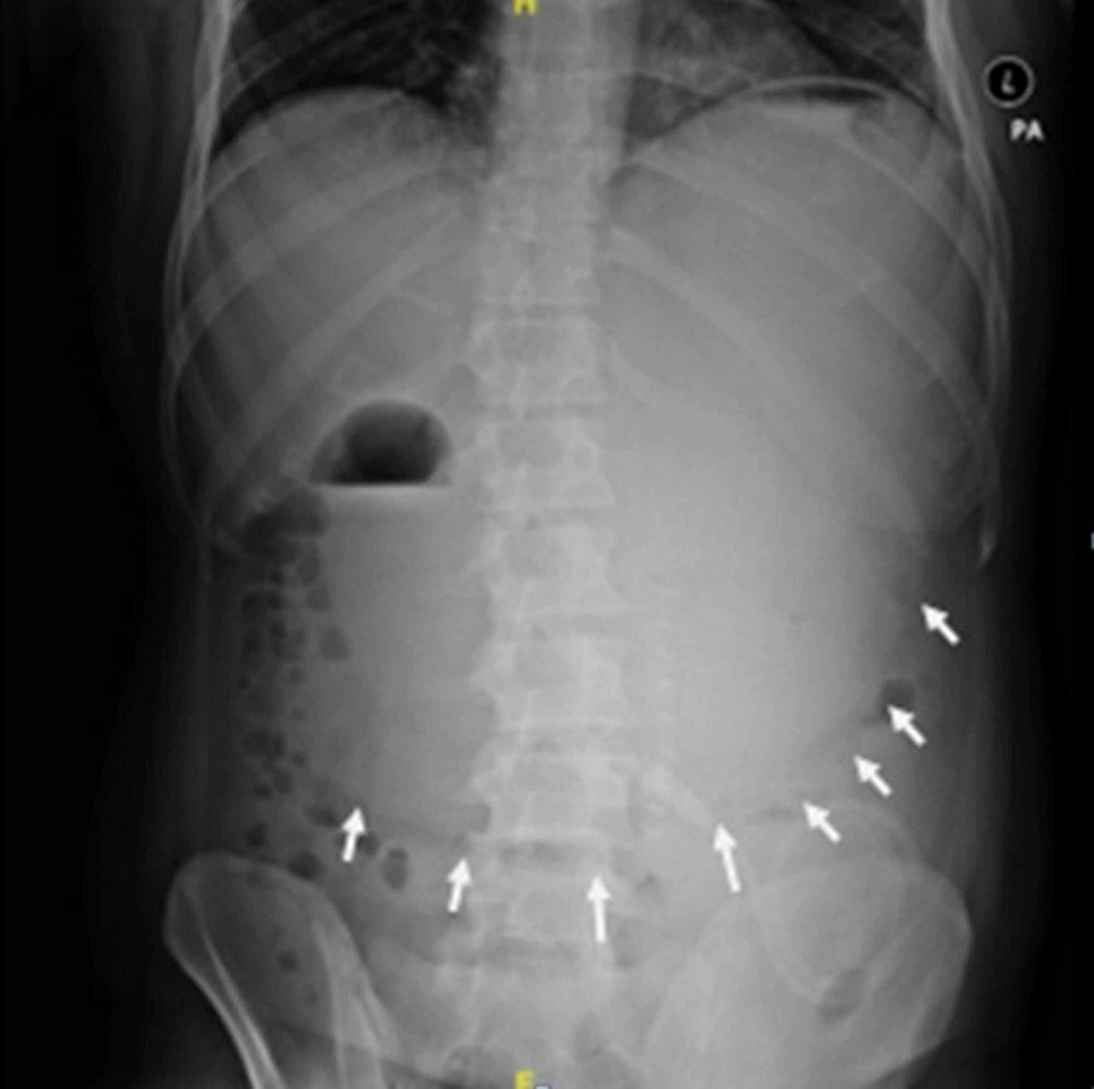
X-ray of the abdomen showing a dilated stomach (arrows are depicting greater curvature of the stomach).

The patient started having abdominal distention from the second postoperative day onward, and it was gradually progressive, although the patient did not have any vomiting episodes. X-ray of the abdomen was performed, which showed massive dilatation of the stomach (Figure 1).

Hence, the patient was re-explored. Intraoperatively, there was gross dilatation of the stomach, duodenum, and jejunum, with kinking at the previous FJ site. The jejunostomy tube was dislodged and the dilated stomach decompressed, passing the same tube retrograde, and a new FJ was performed distally.

**Figure 2:**
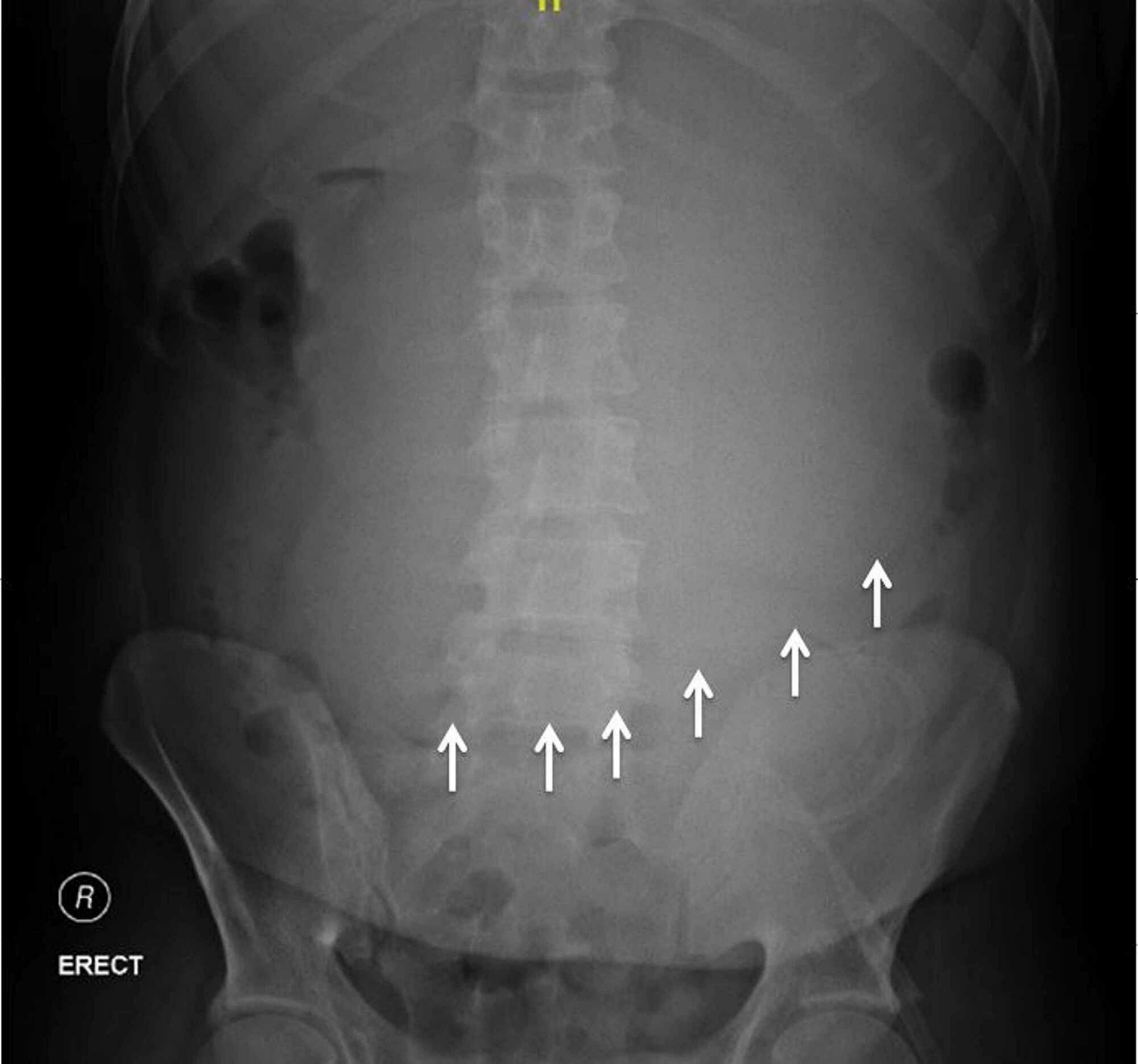
X-ray of the abdomen showing a dilated stomach (arrows).

The next day, the patient had further abdominal distension, tachypnea, and tachycardia. X-ray of the abdomen was repeated, which still showed that the stomach was dilated (Figure 2).

**Figure 3:**
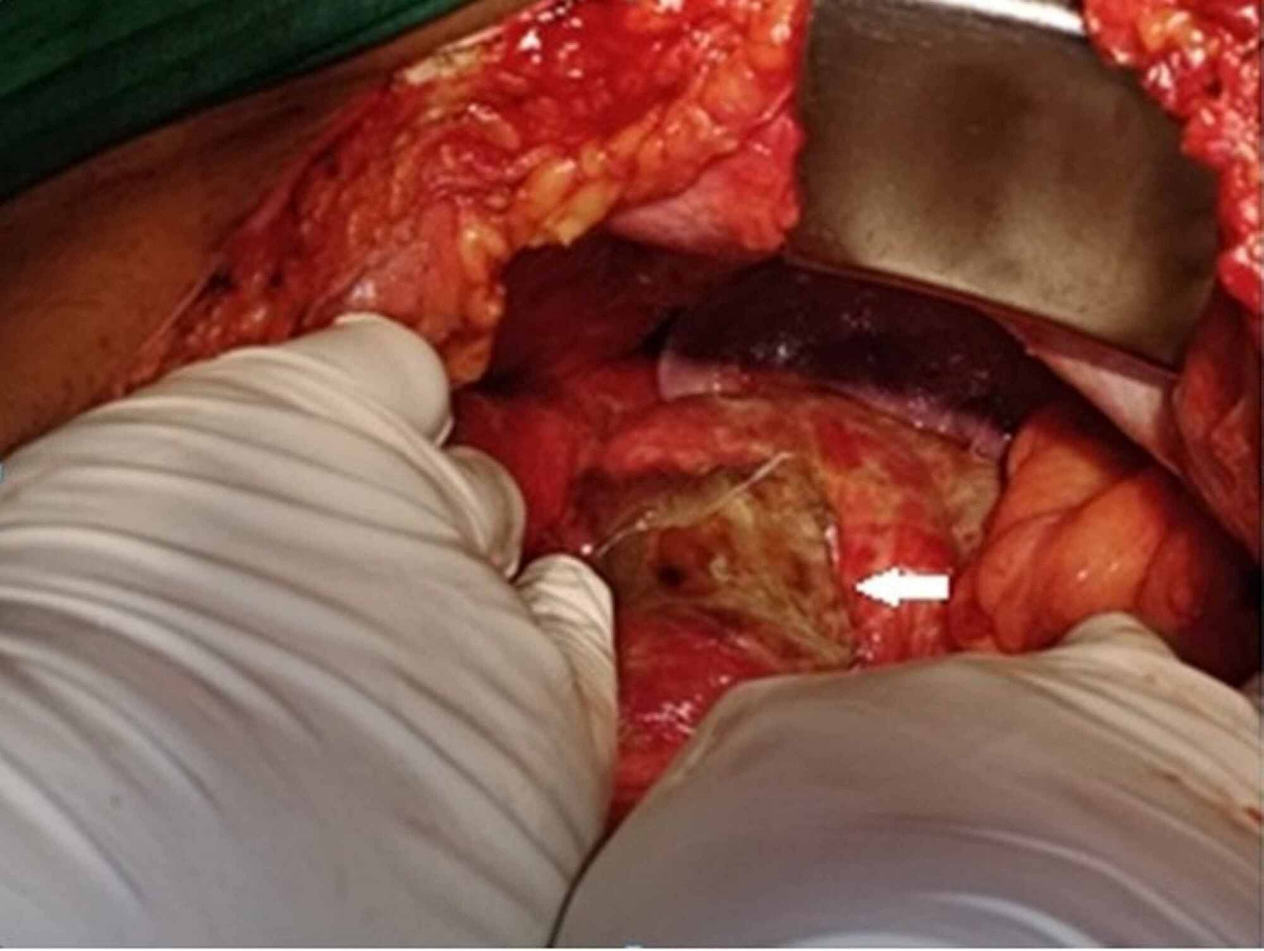
Intraoperative image showing perforation of the stomach at the fundus (arrow).

The patient was re-explored, and this time the stomach was still enlarged, free fluid was present in the abdomen, and there was a perforation of size 3 x 4 cm in the fundus of the stomach (Figure 3).

Perforation was closed primarily with an omental patch. Tube gastrostomy (TG) was performed for drainage purposes. Postoperatively, feeding was started through the FJ tube. Slowly over the next two weeks, the patient improved and was planned radiotherapy for the cricopharyngeal growth. The TG was removed after six weeks.

## Discussion

Duplay, in 1833, was the first one to describe AMGD [1]. Exact etiology is unknown, but it can occur following various medical or surgical conditions [2,3]. The conditions that can lead to AMGD are psychogenic polyphagia, diabetes mellitus, trauma, electrolyte disturbances, gastric volvulus, and binge eating disorder such as anorexia nervosa and bulimia nervosa [4-6]. Most of the cases are reported as a postoperative complication.

Malnutrition is a common problem in patients with upper gastrointestinal malignancies. Patients with cricopharyngeal malignancies also develop malnutrition due to absolute dysphasia, and FJ is a common procedure performed in such patients. FJ can be performed after the completion of a definitive resection procedure or before the neoadjuvant chemoradiotherapy in patients who develop absolute dysphagia or in patients with unresectable cancer.

FJ may lead to complications such as dislodgement, clogging, leakage, bleeding, and small bowel obstruction [7]. A study conducted by Kitagawa et al. had found that 17% of the patients who underwent thoracoscopic esophagectomy developed small bowel obstruction following FJ [8]. There is a literature report in which a patient had undergone FJ for lower-third esophageal carcinoma and later developed intussusception at the FJ site [9].

The pathophysiology of the disease is precisely not known, but there are theories proposed [6,10]. AMGD following closed-loop obstruction of the stomach is very rare. There is a single case report in the literature in which AMGD was reported following closed-loop obstruction of the stomach [11]. Once a certain degree of stomach distention occurs, it is self-perpetuated by increasing its angulation with the duodenum, which follows an increase in gastric distention [12]. Our patient had developed AMGD due to closed-loop obstruction of the stomach following FJ due to the kinking of the FJ site.

The occurrence of gastric perforation in patients with AMGD is rare. In the experimental studies, it has been found that there will be development of tears in the mucus membrane when the stomach is distended with 4 liters of fluid [13]. Hence, before perforation develops, the stomach should be filled beyond 4 liters. It is well known that the stomach is very resistant to ischemia due to its rich blood supply and its extensive intramural anastomosis. Gastric ischemia and necrosis occur when the stomach blood supply is compromised. Whenever the intragastric pressure increases more than 30 cm of water, there will be decreased intramural blood flow and ischemia and necrosis can occur [14]. The most common site of perforation is along the greater curvature and fundus of the stomach. In our patient, we found that the stomach was distended with four and a half liters of fluid, and the perforation was located at the fundus of the stomach.

Clinically, more than 90% of patients will have vomiting [15]. Few patients will not develop vomiting; instead, they are incapable of vomiting. This is due to the occlusion of the gastroesophageal junction as a result of the distended fundus. This leads to an increase in the esophagus’s angulation against the right crus of the diaphragm [16]. Patients develop abdominal pain or will have a distended abdomen, but pain may be mild in intensity [15]. On physical examination, the abdomen will be distended and may be tender. The stomach may be so enlarged that it may be palpable, and succussion splash may be present. Our patient had abdominal distention and pain but did not develop vomiting. The stomach was hugely distended and palpable, and succussion splash was not able to elicit due to pain.

A plain X-ray of the abdomen may show the fluid level with a grossly distended stomach [2]. A computed tomography (CT) scan of the abdomen is most useful for the diagnosis. It demonstrates gastric distention. It will also show the presence of any other mechanical cause of the obstruction. In mentally disabled patients, binge eating may precipitate the superior mesenteric artery (SMA) syndrome, demonstrated by CT scan [17]. Endoscopy is performed to rule out any mechanical causes of obstruction. It also shows the distended stomach, mucosal ulceration, perforation, or any other ischemic changes. In our patient, we could not perform the endoscopy and could not negotiate NGT across the growth, as it was completely obstructing the lumen of the pharynx. We could not perform a CT scan as the patient was not stable and had tachypnea and tachycardia. Hence, the patient was directly taken for laparotomy after X-ray and ultrasonography of the abdomen.

Treatment of AMGD should be started with adequate fluid resuscitation and NGT insertion for stomach decompression. Partial decompression also reduces the pressure and the chance of perforation. Immediate surgery is the option in case of suspected perforation as carries high morbidity and mortality. The surgical treatment of AMGD includes gastric decompression and partial/total gastrectomy. In our case, the patient had gastric perforation near the stomach’s fundus as a result of acute dilatation. TG was performed for gastric drainage and perforation was repaired primarily with an omental patch. The previously performed FJ was used for feeding purposes.

## Conclusions

AMGD with gastric perforation as a result of closed-loop obstruction of the stomach following FJ is very rare. The treating surgeon should be aware of such complications following FJ, particularly in patients who have proximal obstructive growth. A high index of suspicion of AMGD is necessary for patients who have upper abdominal distention following FJ, as it is a rapidly progressive and potentially fatal condition. Endoscopy and CT scans are helpful for the diagnosis. Prompt surgical treatment is required for patients with AMGD who develop complications as it carries a high mortality. The routine practice of FJ in patients with obstructive growth has to be re-evaluated. To the best of our knowledge, this is the first case of AMGD with gastric perforation as a result of the closed-loop obstruction of the stomach due to a kink at the FJ site in a patient with cricopharyngeal carcinoma.
